# Associations of Physical Activity and Sedentary Behavior With Adolescent Academic Achievement

**DOI:** 10.1111/jora.12203

**Published:** 2015-03-23

**Authors:** Marko T. Kantomaa, Emmanuel Stamatakis, Anna Kankaanpää, Eero Kajantie, Anja Taanila, Tuija Tammelin

**Affiliations:** ^1^ LIKES – Research Center for Sport and Health Sciences; ^2^ Imperial College London; ^3^ University of Sydney; ^4^ University College London; ^5^ National Institute for Health and Welfare; ^6^ Helsinki University Central Hospital and University of Helsinki; ^7^ Oulu University Hospital and University of Oulu; ^8^ University of Oulu

## Abstract

The Northern Finland Birth Cohort 1986 (*N *=* *8,061) was used to investigated the joint associations of physical activity and sedentary behavior with academic achievement. Logistic regression analysis was used to evaluate how classes formed by latent class analysis (LCA) according to overall physical activity, sports club membership, viewing TV, using a computer, reading books and magazines, other sedentary activities, and sleep were associated with grade‐point average. When adjusted for gender, self‐rated health, and mother's education, physically active adolescents and generally active adolescents were about twice as likely to have high grade‐point average compared with sedentary TV viewers.

Only one‐third of youth worldwide are estimated to be sufficiently physically active (Ekelund, Tomkinson, & Armstrong, 2011), whereas sedentary behavior has increased during the last few decades; today, young people are spending 4–8 hr sedentary per day (Pate, Mitchell, Byun, & Dowda, 2011). Besides its well‐known benefits on both physical and mental health (Mountjoy et al., 2011), adequate physical activity may also benefit academic achievement in children and youth (Burkhalter & Hillman, 2011; Rasberry et al., 2011; Singh, Uijtdewilligen, Twisk, van Mechelen, & Chinapaw, 2012). Physical activity may enhance psychological functioning (Chaddock‐Heyman et al., 2013; Demirakca et al., 2014; Kalak et al., 2012), attention span and concentration (Coe, Pivarnik, Womack, Reeves, & Malina, 2006), self‐concept and academic self‐confidence (Coatsworth & Conroy, 2007), and, therefore, academic achievement. In addition, higher levels of aerobic fitness have been linked to learning and memory, especially in challenging learning situations (Raine et al., 2013).

In contrast, the increasing level of sedentary behavior has recently raised concerns as a growing education and public health problem (Burkhalter & Hillman, 2011; Schmidt & Vandewater, 2008). For example, media use (Mössle, Kleimann, Rehbein, & Pfeiffer, 2010; Sharif & Sargent, 2006), especially time spent viewing TV (Ennemoser & Schneider, 2007; Espinoza, 2009; Johnson, Cohen, Kasen, & Brook, 2007; Shin, 2004), playing video games (Jackson, von Eye, Witt, Zhao, & Fitzgerald, 2011), and using the Internet (Kim & So, 2012), has been linked to poor academic achievement in children and youth. Excessive screen time may displace comparable activities involving learning opportunities (e.g., doing homework and reading books), lead to mental idleness and passivity (especially TV viewing), and increase impulsive behavior, leading to attention difficulties and academic underachievement (Shin, 2004). Sensation seeking incompatible with concentrated efforts such as reading and writing, learning difficulties, negative attitudes toward school, substance use, and behavioral problems may also explain the association between media use and academic achievement (Johnson et al., 2007; Sharif, Wills, & Sargent, 2010).

Still, not all sedentary behavior is bad for academic achievement; for example, educational TV viewing (Kirkorian, Wartella, & Anderson, 2008; Schmidt & Vandewater, 2008), computer use (Subrahmanyam, Kraut, Greenfield, & Gross, n.d.), and reading books and newspapers (Sullivan & Brown, 2013) have been associated with favorable academic achievement. Today, more traditional forms of sedentary behavior, such as reading books, watching TV, and listening to radio, are intersecting with digital media and online communication (Jenkins, 2009), being a pervasive part of the everyday lives of youth (Ito, 2013). However, adolescents may differ vastly according to the amount they engage in and the way they combine, shape, and structure their physical activity and sedentary behavior in everyday life, which may hold the key to the prevention of the detrimental effects of sedentary behavior, and to the promotion of the benefits of physical activity on learning and academic achievement. To our knowledge, no previous studies have investigated the joint association of physical activity and sedentary behavior with adolescent academic achievement.

This cross‐sectional study investigated the joint association of physical activity, sports club membership, TV viewing, reading books or magazines, playing or working on a computer and playing video games, and other sedentary activities with academic achievement in adolescence. We hypothesized that a high level of physical activity, combined with a reasonable time spent in sedentary behavior, is positively associated with academic achievement.

## Method

### Participants

The study sample consisted of a prospective mother–child birth cohort, the Northern Finland Birth Cohort 1986 (NFBC 1986), which at the baseline was composed of 9,432 infants who were born alive and whose expected date of birth was between July 1, 1985, and June 30, 1986, in the two northernmost provinces of Finland, Oulu and Lapland (Järvelin, Hartikainen‐Sorri, & Rantakallio, 1993). In 2001–2002, at the age of 15–16 years (hereafter referred to as “16 years”), adolescents were sent a postal questionnaire that included questions about their physical activity and sedentary behavior (response rate 80%, *N *=* *7,344). Information on academic achievement at the age of 16 was obtained from the National Application Register for Upper Secondary Education, which contains information on all adolescents in Finland. The present analysis included children with complete information on academic achievement at the age of 16; it excluded children with parental‐reported general learning disability, leaving a final study group of 8,061 children (4,126 boys and 3,935 girls). Informed consent was obtained from all participants and their parents, and the research protocol was approved by the Ethics Committee of Northern Ostrobothnia Hospital District.

### Academic Achievement

Information on academic achievement was provided by the National Application Register for Upper Secondary Education, Finland, based on nationally comparable grades of the final assessment of basic education. The grades refer to a numerical assessment on a scale of 4–10, where 4 denotes a failure (US grade F) and 10 denotes excellent knowledge and skills (US grade A). They describe the level of performance in relation to the objectives of basic education at the end of Grade 9 (age 16). The grade‐point average (GPA) was calculated as a measure of academic achievement, based on grades obtained in the following school subjects: mother tongue (in most cases Finnish or Swedish), first foreign language (started in Grade 3), second national language (started in Grade 7), mathematics, biology, geography, physics, chemistry, religion or ethics, history, music, visual arts, physical education, crafts, and home economics. High academic achievement was defined as GPA ≥ 8.5 (the highest quartile of boys and girls) on a scale of 4.0–10.0. In Finnish basic education, an assessment grade of 8 denotes good, 9 denotes very good, and 10 denotes excellent performance. Finnish GPA 5.0–5.9 equals to 1.0 (D) in US GPA, 6.0–6.9 equals 2.0 (C), 7.0–8.9 equals 3.0 (B), and 9.0–10.0 equals 4.0 (A).

### Physical Activity and Sedentary Behavior

Self‐reported physical activity outside school hours was evaluated separately for moderate‐to‐vigorous physical activity and light physical activity at the age of 16 by asking participants, “How many hours a week all together do you participate in (a) brisk and (b) light physical activity outside school hours?” In the questionnaire, the term *brisk* was defined as physical activity causing at least some sweating and shortness of breath (here referred to as moderate‐to‐vigorous intensity physical activity), while the term *light physical activity* was defined as causing no sweating or shortness of breath. In addition, the adolescents were asked about their time spent daily in physically active commutes to and from school. The response alternatives (*not at all, less than 20 min, 20–39 min, 40–59 min,* and *at least 1 hr per day*) were multiplied by five (5 school days a week) to correspond to 0, 1, 2.5, 3.75, and 5 hr per week (Tammelin, Ekelund, Remes, & Näyhä, 2007). The physical activity level was converted into metabolic equivalent of task (MET) hours per week, based on the intensity and the volume of physical activity engaged in outside school hours, including commuting to and from school (in this study, referred to as *physical activity*). In the calculations, a MET intensity value of three METs was used for light physical activity, five METs for brisk physical activity, and four METs for commuting physical activity (Ainsworth et al., 2011). MET hours per week were further divided into gender‐specific thirds: (1) high (the highest tertile), (2) average (the middle tertile), and (3) low (the lowest tertile). These physical activity questions have good test–retest reliability when administered to Finnish adolescents aged 15–16 years (Tammelin et al., 2007). The intraclass correlation coefficient for physical activity levels described in terms of quintile categories of MET hours per week was .70 (95% confidence interval .58–.80), and the proportion of subjects who were classified in exactly the same category, or next to the same category, in two different tests was 86%.

Participants were also asked about membership and participation in training sessions of a sports club. For the analyses, the adolescents were categorized as sports club members if they reported being members of a sports club, regardless of their participation in the training sessions.

Self‐reported sedentary behavior was evaluated by asking the participants, “How many hours a day on average do you spend in the following activities outside school hours: (1) TV viewing, (2) reading books or magazines, (3) playing or working on a computer and playing video games, and (4) on other sedentary activities?” (Tammelin et al., 2007). Adolescents were classified into three groups according to the time spent on these activities: (1) *<1 hr per day*, (2) *1–2 hr per day*, (3) *>2 hr per day* (Tammelin et al., 2007). Quantity of sleep at the age of 16 years was evaluated in the postal questionnaire by asking the adolescents how many hours on average they spent sleeping per day and was categorized as: (1) *<8 hr per day*, (2) *8 hr per day*, and (3) *>8 hr per day*.

### Potential Covariates

Potential covariates were selected based on our subject matter knowledge (e.g., Kantomaa et al., 2013) and statistical testing. As we had complete information on education level from larger number of mothers compared to fathers, we selected maternal education in the present models. The mother's highest level of education when the adolescent was 16 years was ascertained from the parents. The variable was categorized according to educational level categories used by the International Standard Classification of Education: (1) basic education, lasting 9 years or less; (2) upper secondary education, lasting 10–12 years; and (3) tertiary education, lasting ≥13 years (Statistics Finland, 2001).

Self‐rated overall health was measured by asking, “How would you describe your health at the moment?” The response alternatives were as follows: (1) *very poor*, (2) *poor*, (3) *fair*, (4) *good*, and (5) *very good*.

### Statistical Analyses

Multivariable logistic regression analysis was used to evaluate how classes formed by latent class analysis (LCA), according to self‐reported physical activity, sports club membership duration, and sedentary behaviors, were associated with high GPA. The number of latent classes was approximated by using Akaike's information criterion, Bayesian information criterion (BIC), and sample‐size adjusted BIC. For LCA models with categorical outcomes, the adjusted BIC correctly identifies the number of classes more consistently when compared to other information criteria (Nylund, Asparouhov, & Muthén, 2007). The statistical tests Vuong‐Lo‐Mendell‐Rubin likelihood ratio test, Lo‐Mendell‐Rubin adjusted likelihood ratio test, and parametric bootstrapped likelihood ratio test (BLRT) were applied for determining the number of clusters as well. Entropy was used to evaluate the classification quality. For the further analysis, subjects were classified into their most likely classes. The results of the regression analyses are presented with odds ratios (OR) and 95% confidence intervals [95% CI]. In the multivariable models, the variables were adjusted for gender, self‐rated health, and mother's highest level of education. Full information maximum likelihood estimation with robust standard errors was used under the assumption of data missing at random. The SPSS^®^ 19.0 (IBM Corporation, Armonk, NY, United States) for Windows statistical package (SPSS, 2010) was used for calculating the descriptive statistics and the Mplus statistical package (version 7) (Muthén & Muthén, 2012) was used for latent class analysis (LCA) and multivariable logistic regression analyses.

## Results

At the age of 16, girls (*M *=* *8.1, *SD* = 0.8) had higher academic achievement levels, as indicated by GPA, compared with boys (*M *=* *7.5, *SD* = 0.9), *p *<* *.001. The mean metabolic equivalent of task (MET) hours per week were 32.8 (*SD* = 17.9) for boys and 28.7 (*SD* = 15.5) for girls, *p *<* *.001. The gender‐specific distributions of categorized teacher‐reported academic achievement (GPA), self‐reported physical activity (MET hours per week) and sedentary behavior (TV viewing, reading books or magazines, playing or working on a computer and playing video games, and other sedentary activities), and all contextual variables from the original data are presented in Table 1.

**Table 1:** Characteristics of the Northern Finland Birth Cohort 1986, 2001–2002

Characteristics	Males (*n *=* *4,126)	Females (*n *=* *3,935)	All (*N *=* *8,061)	*p*‐*Value* a
	*n* (%)	*n* (%)	*n* (%)	
Academic achievement^b^	4,065	3,902	7,967	
Average or lower	3,488 (85.8)	2,510 (64.3)	5,998 (5.3)	
Good	577 (14.2)	1,392 (35.7)	1,969 (24.7)	<.001
Physical activity^c^	3,093	3,305	6,398	
Low	871 (28.2)	1,116 (33.8)	1,987 (31.1)	
Average	953 (30.8)	1,227 (37.1)	2,180 (34.1)	
High	1,269 (41.0)	962 (29.1)	2,231 (34.9)	<.001
Sports club membership	3,122	3,336	6,458	
No	2,103 (67.4)	2,491 (74.7)	4,594 (71.1)	
Yes	1,019 (32.6)	845 (25.3)	1,864 (28.9)	<.001
TV viewing	2,956	3,216	6,172	
<1 hr/day	395 (13.4)	445 (13.8)	840 (13.6)	
1–2 hr/day	1,225 (41.4)	1,442 (44.8)	2,667 (43.2)	
>2 hr/day	1,336 (45.2)	1,329 (41.3)	2,665 (43.2)	.008
Computer use/video games	2,995	3,307	6,302	
<1 hr/day	583 (19.5)	2,011 (60.8)	2,594 (32.2)	
1–2 hr/day	1,788 (59.7)	1,208 (36.5)	2,996 (37.2)	
>2 hr/day	624 (20.8)	88 (2.7)	712 (8.8)	<.001
Reading books or magazines	3,098	3,321	6,419	
<1 hr/day	1,054 (34.0)	741 (22.3)	1,795 (28.0)	
1–2 hr/day	1,906 (61.5)	2,294 (69.1)	4,200 (65.4)	
>2 hr/day	138 (4.5)	286 (8.6)	424 (6.6)	<.001
Other sedentary behavior	2,840	3,016	5,856	
<1 hr/day	866 (30.5)	668 (22.1)	1,534 (26.2)	
1–2 hr/day	1641 (57.8)	1,761 (58.4)	3,402 (58.1)	
>2 hr/day	333 (11.7)	587 (19.5)	920 (15.7)	<.001
Sleeping	3,109	3,097	6,454	
<8 hr/day	510 (16.4)	809 (24.2)	1,319 (20.4)	
8 hr/day	1,290 (41.5)	1,482 (44.3)	2,772 (43.0)	
>8 hr/day	1,309 (42.1)	1,054 (31.5)	2,363 (36.6)	<.001
Mother's education	3,104	3,097	6,201	
Basic/upper secondary	2,691 (86.7)	2,698 (87.1)	5,389 (86.9)	
Higher	413 (13.3)	399 (12.9)	812 (13.1)	.622
Self‐rated health	3,134	3,357	6,520	
Very poor	5 (0.2)	3 (0.1)	8 (0.1)	
Poor	23 (0.7)	38 (1.1)	61 (0.9)	
Fair	423 (13.5)	527 (15.7)	955 (14.7)	
Good	1,751 (55.9)	2,119 (63.1)	3,889 (59.7)	
Very good	932 (29.7)	670 (20.0)	1,607 (24.6)	<.001

*Note*

GPA = grade‐point average.

a *p*‐Values for the gender differences (Pearson's chi‐squared test).

b GPA (scale 4.0–10.0) included mother tongue (in most cases, Finnish or Swedish), first foreign language (started at Grade 3), second national language (started at Grade 7), mathematics, biology, geography, physics, chemistry, religion or ethics, history, music, visual arts, physical education, crafts, and home economics. Good academic achievement was classified as GPA ≥ 8.5.

c The physical activity level was defined as metabolic equivalent hours per week based on the intensity and volume of physical activity engaged in outside school hours, including commuting to and from school, and was divided into gender‐specific thirds: (1) high (the highest tertile), (2) average (the middle tertile), and (3) low (the lowest tertile).

High levels of physical activity (OR = 1.58, 95% CI [1.36, 1.84]) and sports club membership (OR = 1.85, 95% CI [1.64, 2.16]) were associated with good academic achievement at the age of 16 (Table 2). Furthermore, reasonable time (<1 hr per day vs. >2 hr per day) spent viewing TV (OR = 1.87, 95% CI [1.56, 2.23]) and playing or working on a computer and playing video games (OR = 1.36, 95% CI [1.06, 1.73]) were associated with good academic achievement, compared with higher amounts of sedentary behaviors. In addition, spending 1–2 hr per day in other sedentary behaviors (OR = 1.35, 95% CI [1.14, 1.61]) and sleeping for 8 hr per day (OR = 1.48, 95% CI [1.26, 1.74]), were associated with good academic achievement, compared with higher levels of other sedentary behaviors and sleeping for less than 8 hr per day, respectively.

**Table 2:** Logistic Regression of Good Academic Achievement and the Level of Physical Activity and Sedentary Behavior

	*n*	Good Academic Achievement^a^	
		%	Adjusted^b^ OR [95% CI]
Physical activity^c^	6,398		
Low	1,987	21.9	1.00
Average	2,180	29.6	1.44 [1.24, 1.67]
High	2,231	30.3	1.58 [1.36, 1.84]
Sports club membership	6,458		
No	4,594	71.1	1.00
Yes	1,864	28.9	1.75 [1.54, 1.99]
TV viewing	6,172		
>2 hr/day	2,665	21.5	1.00
1–2 hr/day	2,667	32.7	1.68 [1.47, 1.90]
<1 hr/day	840	35.6	1.87 [1.56, 2.23]
Computer use/video games	6,302		
>2 hr/day	2,594	16.2	1.00
1–2 hr/day	2,996	23.7	1.11 [.88, 1.40]
<1 hr/day	712	35.3	1.36 [1.06, 1.73]
Reading books or magazines	6,419		
>2 hr/day	424	31.2	1.00
1–2 hr/day	4,200	29.2	1.05 [.83, 1.32]
<1 hr/day	1,795	22.4	.85 [.66, 1.09]
Other sedentary behavior	5,856		
>2 hr/day	920	25.5	1.00
1–2 hr/day	3,402	29.6	1.35 [1.14, 1.61]
<1 hr/day	1,534	26.6	1.25 [1.03, 1.53]
Sleeping	6,454		
<8 hr/day	1,319	24.0	1.00
8 hr/day	2,772	31.4	1.48 [1.26, 1.74]
>8 hr/day	2,363	24.8	1.16 [.98, 1.37]

*Note*

GPA = grade‐point average; OR = odds ratios; 95% CI = 95% confidence intervals.

(*N* = 8,061).

a GPA (scale 4.0–10.0) included mother tongue (in most cases, Finnish or Swedish), first foreign language (started at Grade 3), second national language (started at Grade 7), mathematics, biology, geography, physics, chemistry, religion or ethics, history, music, visual arts, physical education, crafts, and home economics. Good academic achievement was classified as GPA ≥ 8.5.

b Adjusted for gender, self‐rated health, and mother's level of education.

c The physical activity level was defined as metabolic equivalent hours per week based on the intensity and volume of physical activity engaged in outside school hours, including commuting to and from school, and was divided into gender‐specific thirds: (1) high (the highest tertile), (2) average (the middle tertile), and (3) low (the lowest tertile).

**Figure 1:**
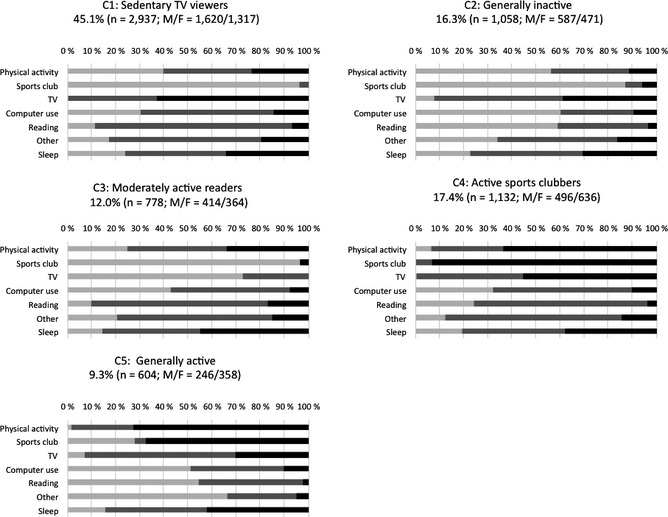
Characteristics of the classes (C1–C5) formed by physical activity and different forms of sedentary behavior at the age of 16 years (*n *=* *6,509). The colors denote percentages of the class sample in low (light gray), medium (dark gray), and high (black) levels of activity. M = male; F = female.

Latent class analysis models were fitted to the seven physical activity and sedentary behaviors reported above. Information criteria and statistical tests, except for the BLRT, indicated that the number of classes would be 4–6 (Table 3). Based on the lowest value of the adjusted BIC, the five‐class solution was chosen. Entropy for this solution was .60. Figure 1 presents the response probability profiles for each of the five classes (C1–C5). The first class (C1: sedentary TV viewers) was characterized by watching TV for more time, spending less time in other sedentary activities, having low overall physical activity, and not being members of a sports club. Adolescents in class 2 (C2: generally inactive) had low overall physical activity, were rarely members of a sports club, and spent little time in sedentary activities. The third class (C3: moderately active readers) was characterized by having a moderate level of overall physical activity, rarely being members of a sports club, spending a fair bit of time reading books and magazines, but only a little time on other sedentary behavior, especially watching TV. Adolescents in class 4 (C4: active sports clubbers) had a high overall level of physical activity, were often members of a sports club, and spent a fair amount of time using various media. The fifth class (C5: generally active) were physically active, were often members of a sports club, and spent little time in sedentary activities. Adolescents in different classes did not have significant differences according to quantity of sleep (Figure 1).

**Table 3:** Classes Identified Through Latent Class Analysis (LCA) with Goodness‐of‐Fit Statistics

Number of classes	AIC	BIC	Adjusted BIC	VLMR	LMR	BLRT	Entropy
2	81,895	82,092	82,000	<.001	<.001	<.001	.91
3	81,673	81,972	81,832	.0003	.0003	<.001	.59
4	81,515	81,915	81,728	.0007	.0008	<.001	.57
5	81,444	81,945	81,710	1.0000	1.0000	<.001	.60
6	81,413	82,017	81,734	1.0000	1.0000	<.001	.56

*Note*

AIC = Akaike's information criterion; BIC = Bayesian information criterion; VLMR = Vuong‐Lo‐Mendell‐Rubin likelihood ratio test; LMR = Lo‐Mendell‐Rubin adjusted likelihood ratio test; BLRT = parametric bootstrapped likelihood ratio test.

(*n* = 6,509).

Adolescents in classes C3 (moderately active readers), C4 (active sports clubbers), and C5 (generally active) more commonly (35% vs. 22%) had high GPA compared with adolescents in classes C1 (sedentary TV viewers) and C2 (generally inactive) (Table 4). When adjusted for gender, self‐rated health, and mother's education, generally active adolescents (OR = 2.02, 95% CI [1.65, 2.48]) and active sports clubbers (OR = 1.98, 95% CI [1.68, 2.33]) were about twice as likely to have high GPA as compared with sedentary TV viewers. In addition, moderately active readers (OR = 1.81, 95% CI [1.51, 2.18]) were almost twice as likely to have high GPA as compared with sedentary TV viewers. There was not a statistically significant difference according to academic achievement between sedentary TV viewers and generally inactive adolescents (Table 4).

**Table 4:** Logistic Regression of Good Academic Achievement and the Classes Identified Through Latent Class Analysis (LCA)

	*n*	Good Academic Achievement^a^		
		%	Unadjusted OR [95% CI]	Adjusted^b^ OR [95% CI]
Class 1 (sedentary TV viewers)	2,937	22.4	1.00	1.00
Class 2 (generally inactive)	1,058	22.3	.99 [.84, 1.18]	1.02 [.85, 1.22]
Class 3 (moderately active readers)	778	35.0	1.87 [1.57, 2.21]	1.81 [1.51, 2.18]
Class 4 (active sports clubbers)	1,132	35.0	1.86 [1.60, 2.16]	1.98 [1.68, 2.33]
Class 5 (generally active)	604	34.7	1.85 [1.53, 2.23]	2.02 [1.65, 2.48]

*Note*

GPA = grade‐point average; OR = odds ratios; 95% CI = 95% confidence intervals.

(*N* = 8,061).

a GPA (scale 4.0–10.0) included mother tongue (in most cases, Finnish or Swedish), first foreign language (started at Grade 3), second national language (started at Grade 7), mathematics, biology, geography, physics, chemistry, religion or ethics, history, music, visual arts, physical education, crafts, and home economics. Good academic achievement was classified as GPA ≥ 8.5.

b Adjusted for gender, self‐rated health, and mother's level of education.

## Discussion

In this study, a high level of physical activity, with a reasonable amount of time spent in sedentary behavior, such as media use, was associated with good academic achievement, whereas a high level of sedentary behavior, especially TV viewing, was associated with lower levels of academic achievement in adolescence.

Our finding of a positive association between physical activity and academic achievement is consistent with previous studies concluding that self‐reported physical activity is associated with good academic achievement in adolescents (Fox, Barr‐Anderson, Neumark‐Sztainer, & Wall, 2010; Kantomaa et al., 2013; Nelson & Gordon‐Larsen, 2006). Physical activity may enhance brain health, structure, and function (Chaddock‐Heyman et al., 2013; Demirakca et al., 2014; Hillman, Erickson, & Kramer, 2008); induce arousal and reduce boredom, leading to increased attention span and concentration (Coe et al., 2006); and improve self‐concept and academic self‐confidence (Coatsworth & Conroy, 2007). Furthermore, physical activity may enhance young people's cognitive functions, such as executive functions and working memory, and, therefore, academic achievement (Castelli, Hillman, Hirsch, Hirsch, & Drollette, 2011; Chaddock, Hillman, Buck, & Cohen, 2011; Davis et al., 2011).

Our results support previous studies reporting that participation in organized physical activity is positively associated with academic achievement (Rasberry et al., 2011). This finding may have special significance in light of the increase in participation in organized youth sports over the past decade (Myer et al., 2011). It has been proposed that participation in organized sports fosters student identification with school and school‐related values, including performing well academically (Fox et al., 2010). Participation in organized physical activities may also develop young people's ability to listen to and follow instructions, to wait for their own turn, and to choose ways of action appropriate for the situation. Furthermore, physical activity may provide young people with opportunities to vent and process their feelings (Kantomaa, Tammelin, Ebeling, & Taanila, 2008). At its best, engaging in organized physical activities develops teamwork skills, self‐direction, and the ability to cooperate with different people (Kantomaa et al., 2008). All of these aspects may contribute to explaining the good academic achievement of adolescents participating in organized physical activities.

Our results are in line with previous findings showing that reasonable time spent in sedentary behavior, such as media use, is positively associated with academic achievement (Schmidt & Vandewater, 2008; Williams, Haertel, Haertel, & Walberg, 1982). However, it seems that the context of the media use and the content delivered by the media are more influential than the media themselves (Borzekowski & Robinson, 2005; Schmidt & Vandewater, 2008). For example, viewing educational TV is positively linked with academic achievement, whereas viewing entertainment TV is negatively linked with achievement (Kirkorian et al., 2008; Schmidt & Vandewater, 2008). Furthermore, a recent study reported that video gaming induces structural brain plasticity, which may benefit cognitive functions such as working memory, behavioral flexibility, attention, and future planning (Kühn, Gleich, Lorenz, Lindenberger, & Gallinat, 2014) and, therefore, academic achievement (Castelli et al., 2011; Chaddock et al., 2011; Davis et al., 2011).

Today, more traditional media, such as books, television, and radio, are intersecting with digital media (Jenkins, 2009), providing young people with more opportunities for online social interaction, problem solving, and self‐directed activity that lead to diverse forms of learning in formal and informal settings (Ito, 2013). The present study demonstrates that, when combined with the various benefits of physical activity on learning and cognitive function, digital and media technology‐mediated learning may provide adolescents with skills and abilities that best benefit academic achievement in today's complex and ubiquitous learning environments. However, it should be pointed out that in 2012 in the United States, for example, 95% of U.S. adolescents reported using the Internet, compared to 87% in 2004, and 73% in 2000 (Madden, Lenhart, Duggan, Cortesi, & Gasser, n.d.), 1 year before the start of the present data collection (2001–2002). Also the use of digital media among adolescents has changed considerably during the past decade, for example, from stationary connections to always‐on (mobile) connections that move with the user throughout the day (Madden et al., n.d.). It is possible that the association between media use and academic achievement would be slightly different in current data. On the other hand, today, increasing mobile connectivity may provide young people with whole new opportunities for combining physically active lifestyle, media use, and technology‐enhanced learning.

Our study was based on a large, unselected population sample with a high participation rate (80%). Information on academic achievement was based on nationally comparable grades of the final assessment of basic education obtained from the National Application Register for Upper Secondary Education, providing a representative and comprehensive estimate of adolescents' academic achievement. In addition, we included various measures of physical activity and sedentary behavior in our models and were able to provide a comprehensive picture of these behaviors within various activity domains. However, we did not have information on the content of the media delivered, which may be crucial for the influence of sedentary behavior on academic achievement. The cross‐sectional setting of the present study does not allow us to draw strong conclusions about causality between physical activity, sedentary behavior, and academic achievement. Furthermore, our study relied on adolescents' self‐reported physical activity and sedentary behavior, which might contain relatively large measurement errors and social desirability bias (Sallis & Saelens, 2000; Shephard, 2003). Finally, the present data was collected in 2001–2002, limiting the interpretation of the results in light of the current social environment with most adolescents having pervasive access to Internet, more commonly through mobile devices (Madden et al., n.d.). Nonetheless, our study sample represents a generation considered “digital natives” (Prensky, 2001) and it may therefore be a reasonable reflection of the younger generation with respect to media use.

The results of the present study form a basis for future research investigating the possible causality between physical activity, sedentary behavior, and academic achievement. It would be useful to investigate the associations in more contemporary cohorts, and within different sociocultural settings. Better identification of mediating and moderating variables would be especially beneficial for physical activity interventions, which could be targeted at improving these behaviors, as well as academic outcomes. It is also possible that the joint association of physical activity and sedentary behavior with academic achievement varies according to the forms of these activities, offering interesting viewpoints for future studies.

In conclusion, a high level of physical activity and a reasonable time spent in sedentary behavior, such as media use, were associated with good academic achievement, whereas a high amount of sedentary behavior, especially TV viewing, was associated with lower levels of academic achievement in adolescence. In today's complex and dynamic learning environments, physical activity combined with reasonable time using multiple media may provide young people with the skills and abilities that will best benefit their academic achievement.
